# One Part Leaky Covers

**DOI:** 10.1007/s44007-026-00242-9

**Published:** 2026-09-07

**Authors:** Renzo Cavalieri, Hannah Markwig, Johannes Schmitt

**Affiliations:** 1 Department of Mathematics, Colorado State University, Weber Building, Fort Collins, CO 80523-1874 USA; 2 Fachbereich Mathematik, Universität Tübingen, Auf der Morgenstelle 10, 72076 Tübingen, Germany; 3 Department Mathematik, ETH Zürich, Rämistrasse 101, CH-8092 Zürich, Switzerland

**Keywords:** k-leaky Hurwitz numbers, Descendant invariants, Tropical covers, Double Ramification cycles

## Abstract

The paper [7] defined a new class of enumerative invariants called *k*-leaky double Hurwitz descendants, generalizing both descendant integrals of double ramification cycles and *k*-leaky double Hurwitz numbers. Here, we focus on the one-part version of these numbers, i.e. when k≥0 and the positive ramification profile is (*d*). We derive recursions and use them to produce explicit formulas and structure results for some infinite families of these numbers.

## Introduction

We begin this introduction by summarizing the original results of this work, which we then comment on in Sect. 1.2. In Sect. 1.3 we expand our discussion to provide an overview of how the problem fits within its broader field of research. While this “onion-style” approach is non-standard, we feel it provides the most efficient access to the contents of this paper for readers that are somewhat familiar with the interactions of tropical and logarithmic geometry in curve counting problems. We tried to write each section in a self-contained way, so readers may read them in whatever order suits them best.

### Results

We study one-part *k*-leaky double Hurwitz numbers with ψ-class insertions $\textsf{H}_g((d, - \boldsymbol{\nu }),$ $\textbf{e})$, with the main goal of exhibiting explicit formulas and unveiling part of the rich combinatorial structure of these enumerative invariants. We will give precise definitions later, but for now it suffices to view $\textsf{H}_g((d, - \boldsymbol{\nu }), \textbf{e})$ as an enumerative invariant for pointed curves of genus *g*; the discrete data $(d, - \boldsymbol{\nu })$, with $\boldsymbol{\nu }= (\nu _1, \ldots , \nu _m)$ a vector of length *m*, and $\textbf{e}$ encodes ramification conditions and ψ-class insertions at the marked points.

The invariants also depend on a leaking parameter $k\in \mathbb {Z}_{\ge 0}$, which is suppressed from the notation. This convention is the one-part chamber with the unique positive ramification condition on the left. Reversing the orientation gives the symmetric one-part-to-the-right convention with k≤0; here we fix the k≥0 convention.

We first consider leaky descendant Hurwitz numbers with genus g=0 and ψ-insertions only at the unique positive marking $p_0$ and at most one other marking, which we choose to be $p_1$. We introduce the notation

1 $$\begin{aligned} J_{e_0,e_1}^m (k,d,\nu _1):=\textsf{H}_0((d, - \boldsymbol{\nu }), (e_0, e_1, 0,\ldots , 0)) \end{aligned}$$

for m≥2, where the discrete data additionally satisfies the balancing condition $d - \sum _{i=1}^m \nu _i = k \cdot (m-1)$. As suggested by the notation, these invariants are independent from the ramification orders at points that do not support a ψ-class, but depend on the length *m* of the partition ${\boldsymbol{\nu }}$.

#### Theorem 1

For $k\in \mathbb {Z}_{\ge 0}$, the following formulas hold for the families of *k*-leaky one-part double Hurwitz descendants defined in (1):

2 $$\begin{aligned} J^m_{e_0,0}(k,d,\nu _1)&= \frac{(m-1)!}{(e_0+1)!} \cdot \prod _{j=e_0+1}^{m-2} \left( d-j\cdot \frac{k}{2}\right) \,, \end{aligned}$$

and

3 $$\begin{aligned} J^m_{0,e_1}(k,d,\nu _1)&= \frac{(m-2)!}{(e_1+1)!} \cdot \Big ( e_1 \cdot \frac{\prod _{j=e_1+1}^{m-1} (d-\nu _1-j\cdot \frac{k}{2}) - \prod _{j=1}^{m-e_1-1} (d-j\cdot \frac{k}{2})}{-\nu _1-e_1\cdot \frac{k}{2}} \nonumber \\&\quad + (m-e_1-1) \cdot \prod _{j=1}^{m-e_1-2} \left( d-j\cdot \frac{k}{2}\right) \Big )\,. \end{aligned}$$

Formula (3) has the following specializations when setting k=0 or $\nu _1=0$:

4 $$\begin{aligned} J^m_{0,e_1}(0,d,\nu _1)&= \frac{(m-2)!}{(e_1+1)!} \cdot \left( e_1 \cdot \frac{d^{m-1-e_1} - (d-\nu _1)^{m-1-e_1}}{\nu _1}+(m-1-e_1)\cdot d^{m-2-e_1}\right) ,\end{aligned}$$

5 $$\begin{aligned} J^m_{0,e_1}(k,d,0)&= \frac{(m-2)!}{(e_1+1)!} \cdot (m-1-e_1) \cdot \left( \sum _{i=1}^{e_1+1} \ \prod _{j=0}^{m-e_1-3}\left( d-(i+j)\cdot \frac{k}{2}\right) \right) \,. \end{aligned}$$

We record these specializations because of their simpler shape: the case k=0 in (4), where $d = \sum _{i=1}^m \nu _i$, gives a closed formula for classical (non-leaky) one-part double Hurwitz descendants with a single insertion $\psi _1^{e_1}$ at the marking with negative ramification $-\nu _1$ (for $e_1=0$ it recovers the one-part double Hurwitz numbers $(m-1)! \cdot d^{m-2}$ of [11]), and both specializations served as stepping stones towards discovering the general formula (3), see Sect. 1.2.

Still for g=0 and arbitrary ${\textbf {e}}$, the piecewise polynomiality results of [7] imply that the invariant $\textsf{H}_0((d, - \boldsymbol{\nu }), \textbf{e})$ is a polynomial of degree $m-2-|{\textbf {e}}|$ on the chamber containing the one-part data. In Proposition 16 we give an independent, recursive proof of this polynomiality, which shows moreover that the polynomial only depends on *d*, *k* and the variables $\nu _i$ associated to markings with non-trivial ψ-insertions (i.e. indices $1 \le i \le m$ with $e_i > 0$).

In genus one, we prove a formula that describes one part leaky Hurwitz numbers (with no descendant insertions) for arbitrary non-negative values of *k*.

#### Theorem 2

For $k\in \mathbb {Z}_{\ge 0}$, the following formula holds:

6 $$\begin{aligned}&\textsf{H}_1((d, - \boldsymbol{\nu }), {\textbf {0}})=\nonumber \\  &\frac{(m+1)!}{24} \cdot \prod _{j=2}^m \left( d - j\cdot \frac{k}{2}\right) \cdot \left[ (d-k)^3 - \left( d-\frac{k}{2}\right) \cdot \left( 1+ 2\cdot \sum _{s<t}(\nu _s+k)(\nu _t+k)\right) \right] . \end{aligned}$$

In arbitrary genus g≥0 we have a complete understanding of the k=0 specialization of the *k*-leaky double Hurwitz descendants with all ψ-insertions at marking $p_0$; the structure we observe naturally generalizes and interpolates between the case $e_0=0$ treated in [11, Theorem 3.1] and the case $e_0=2g-2+m$ from [3, Theorem 1].

#### Theorem 3

For g,m≥0 with 2g-1+m>0, we have

7 $$\begin{aligned} \textsf{H}_g((d,-\boldsymbol{\nu }), (e_0, 0, \ldots , 0))|_{k=0} = \frac{d^{-e_0}}{(e_0+1)!}\cdot \textsf{H}_g((d,-\boldsymbol{\nu }), ( 0, \ldots , 0))|_{k=0}. \end{aligned}$$

In particular, since [3] provides a formula for descendant invariants, we also obtain explicit formulas for all intermediate invariants. Denote

$$ S(z) = \frac{\sinh (z/2)}{z/2} = \sum _{k \ge 0} \frac{z^{2k}}{2^{2k}(2k+1)!} = 1 + \frac{z^2}{24} + \frac{z^4}{1920} + \frac{z^6}{322560} + \ldots $$

considered as a formal power series in $\mathbb {Q}[z]$. We have

8 $$\begin{aligned} \textsf{H}_g((d,-\boldsymbol{\nu }), (e_0,0,\ldots , 0))|_{k=0} = \frac{(2g-1+m)!}{(e_0+1)!} \cdot d^{2g-2+m-e_0} \cdot [z^{2g}] \frac{\prod _{j=1}^m S(\nu _j z)}{S(z)}\,, \end{aligned}$$

where $[z^{2g}]$ means taking the coefficient of the monomial $z^{2g}$ in the power-series expansion in *z* of the expression that follows.

### Discussion

Leaky descendant Hurwitz numbers, defined in [7, Definition 1.1] are degrees of 0-dimensional classes in the logarithmic Chow ring of $\overline{\mathcal {M}}_{g,n}$, obtained by multiplying a log DR cycle with two distinguished types of classes: ψ-classes pulled-back from $\overline{\mathcal {M}}_{g,n}$ and piecewise polynomial branch classes pulled back from $\textsf{tEx}$ via the tropical branch morphism. Informally, $\textsf{tEx}$ is a cone stack and these piecewise polynomials determine classes of loci of curves that degenerate in a prescribed way; for a precise discussion see [6, Sections 2.7, 2.8]. Consider $\textbf{x} = (d, - \boldsymbol{\nu }) = (d, -\nu _1, \ldots , -\nu _m)$ of length n=m+1, where $d, \nu _1, \ldots , \nu _m > 0$ satisfy

$$\begin{aligned} d - \sum _{i=1}^m \nu _i = k \cdot (2\,g-2+n)\,. \end{aligned}$$

Labeling the marks of $\overline{\mathcal {M}}_{g,n}$ by $p_0, p_1, \ldots , p_m$ and denoting $e = |\textbf{e}|$, we have

9 $$\begin{aligned} \textsf{H}_g((d, - \boldsymbol{\nu }), \textbf{e}) = \int _{{{\,\textrm{logDR}\,}}_g(d, - \boldsymbol{\nu })} \psi _0^{e_0}\cdots \psi _m^{e_m} \cdot {{\,\textrm{branch}\,}}_{2g-3+n-e}\,. \end{aligned}$$

This paper takes the first steps in studying the algebraic and combinatorial structure arising from the dependence of these enumerative invariants on their discrete data. We deem this endeavor interesting for two reasons: first because, when k=0, these invariants naturally interpolate between double Hurwitz numbers and descendant invariants of the DR cycle, two classical families of enumerative invariants that are well-known to have rich structure. Secondly, letting k>0 one can observe interesting parallels between invariants of moduli spaces of maps and of moduli spaces of (pluri)differentials.

The main tool we use to study this structure is a tropical algorithm ([7, Theorem 1.2]) computing leaky descendant Hurwitz numbers as a weighted sum over leaky tropical covers, see Sect. 2.

Such perspective provides a natural recursion among these invariants: truncating all leaky covers before their last vertex and organizing the sums in terms of the type of the last vertex expresses a leaky descendant Hurwitz number as a finite sum of similar invariants with smaller value for the quantity 2g-2+n. This recursion is stated in Lemmas 13, 14, 15 and 17, each time in the generality needed at that point.

While such recursive approach is always available, rather than attempting a general description, we choose to focus our attention on a few infinite families of double leaky Hurwitz descendants where the structure is especially explicit and simple.

Restricting to the one-part chamber (where we have only the point $p_0$ with positive ramification condition) and to k≥0 has two simplifying effects: the recursion only involves connected invariants, and the resulting families of invariants are polynomial, rather than piecewise polynomial.

Limiting the number of ψ-insertions has the effect of limiting the combinatorial possibilities for the cut-off vertex in the tropical leaky covers, which reduces the combinatorial complexity of the recursion.

In genus zero, the last vertex of any tropical leaky cover must have a unique left pointing flag, so all recursive terms have strictly fewer marked points. We focus our attention on invariants with exactly one descendant insertion of arbitrary power. It is convenient to introduce the notation $J_{e_0,e_1}^m$ (1) that allows insertions both on the unique mark with positive profile as well as on a single mark with negative profile in order to state efficiently the recursion; eventually Theorem 1 gives explicit formulas for these invariants in the cases $(e_0, 0)$ and $(0,e_1)$.

Our path towards Theorem 1 went through the following four steps:

1. The tropical algorithm computing the numbers $J_{e_0,e_1}^m$ was implemented based on the software package admcycles [9]. From [7, Theorem 1.3] it follows that $J_{e_0,e_1}^m$ is a polynomial of degree $m-2-e_0-e_1$ in $d, \nu _1, \ldots , \nu _n$, where *k* is then determined as
  $$\begin{aligned} k=(d-\nu _1 - \ldots - \nu _m)/(m-1). \end{aligned}$$
  Thus for any fixed $e_0, e_1, m$, a formula for $J_{e_0,e_1}^m$ can be obtained using multivariate interpolation by evaluating at finitely many points $\textbf{x} = (d, - \boldsymbol{\nu })$.
2. Encouraged by the simple shape of the resulting formulas (for small parameters $e_0, e_1, m$), we identified the recursion for $J_{e_0,e_1}^m$, see Lemma 14. This allows the calculation for larger values of *m*, which would be infeasible to obtain just from the interpolation method of Step 1.
3. In the cases $e_1=0$ or $e_0=0$ presented in Theorem 1, we guessed the shape of the formulas presented there from the computational evidence and some intuition derived from the shape of the recursion in Lemma 14. In the case $e_0=0$, we first found the specializations (4) and (5) before managing to find the general shape (3).
4. Once a candidate formula for $J_{e_0,e_1}^m$ is available, it can be proven to satisfy the recursion derived in Lemma 14 via lengthy, but ultimately manageable algebraic manipulations.

In generalizing the process described above, the bottleneck (for the authors) lies in Step 3, where a general formula must be guessed from limited data. This data is available in many further cases not covered above (e.g. when both $e_0, e_1 > 0$), and we expect that reasonably simple formulas could exist here.

In genus one we restrict our attention to the case $\textbf{e} =0$, where the formula we guessed is shown to satisfy the recursion from Lemma 17 by expressing it as a polynomial in the elementary symmetric functions in the quantities $(\nu _i+k)$. The formula in Theorem 2 is easily shown to be equivalent to

$$\begin{aligned}&\textsf{H}_1((d, - \boldsymbol{\nu }), {\textbf {0}}) =\\  &\frac{(m+1)!}{24} \cdot \left( \left( \sum _{i=1}^{m} (\nu _i + k)^2 - 1 \right) \cdot \prod _{j=1}^{m} \left( d - j\cdot \frac{ k}{2}\right) - k\cdot \frac{\left( d - k\right) ^2}{2} \cdot \prod _{j=2}^{m} \left( d - j\cdot \frac{ k}{2}\right) \right) \,. \end{aligned}$$

which, setting k=0, immediately reduces to the formula for one part genus one double Hurwitz numbers proven in [11].

For arbitrary genus *g*, we set k=0 but allow one descendant insertion of arbitrary degree at the unique point of positive ramification. In this case we have a different recursion at our disposal, consisting of exchanging one ψ-class for a linearly equivalent boundary divisor. In k=0, such operation follows from the existence of a branch morphism from the DR cycle to a (quotient of) Losev-Manin space, as well as known comparison results among the ψ-classes on Losev-Manin, DR and $\overline{\mathcal {M}}_{g,n}$. In [7, Proposition 4.2] the authors show a similar exchanging rule that trades a ψ-class with a boundary divisor plus a multiple of the class $\kappa _1$ restricted to the logarithmic double ramification cycle.

In Sect. 5 we describe the existing computer implementations of formulas for $\textsf{H}_g(\textbf{x}, \textbf{e})$ (and how to access them). The authors encourage interested readers to try their hands at finding new formulas and shedding light on the intriguing structure that can be already observed in these first steps. For example, there seems to be a hypergeometric dependence of these invariants on the parameter *k*, and it would be satisfying to have a conceptual explanation for this phenomenon.

### Context

Hurwitz numbers are classical enumerative invariants counting branched covers of the projective line [12]. The ELSV formula [10] establishes a connection between single Hurwitz numbers and intersection theory on moduli spaces of curves, revealing polynomial structure and links to Gromov–Witten theory.

Double Hurwitz numbers $H_g(\boldsymbol{\mu },\boldsymbol{\nu })$ extend this setting by counting genus *g* covers with specified ramification profiles $\boldsymbol{\mu }$ over 0 and $\boldsymbol{\nu }$ over ∞, with simple branching elsewhere. The seminal work of Goulden, Jackson, and Vakil [11] established that for fixed genus *g*, these numbers are piecewise polynomial in the parts of the partitions $\boldsymbol{\mu }$ and $\boldsymbol{\nu }$. Shadrin, Shapiro, and Vainshtein [15] analyzed the chamber structure of $H_0(\boldsymbol{\mu },\boldsymbol{\nu })$, explicitly describing wall-crossing behavior, while Cavalieri, Johnson, and Markwig [4] developed tropical methods that provided a systematic derivation of wall-crossing formulas in all genera. Johnson [13] employed infinite-wedge Fock space formalism to derive explicit formulas for double Hurwitz numbers with arbitrary ramification profiles, providing another proof of piecewise polynomiality.

A special chamber of polynomiality parameterizes one-part double Hurwitz numbers, where one ramification profile consists of a single part. In genus 0, Goulden-Jackson-Vakil established the formula:

10 $$\begin{aligned} {H_0}((d),\boldsymbol{\nu }) = (n-1)! \cdot d^{n-2} \end{aligned}$$

where $\boldsymbol{\nu }=(a_1,\ldots ,a_n)$ is a partition of degree *d* with *n* parts. For genus g≤5, they compute explicit polynomial expressions representing one part double Hurwitz numbers as the product of a monomial in *d* and even symmetric polynomials in the $\nu _i$’s. They further conjectured an ELSV-type formula for one-part double Hurwitz numbers $H_g((d),\boldsymbol{\nu })$, i.e. a tautological intersection theoretic expression on some compactification of the universal Picard stack of the form:

11 $$\begin{aligned} H_g((d),\boldsymbol{\nu }) = d\cdot r! \int _{\overline{\textrm{Pic}}_{g,n}} \frac{\Lambda _0 -\Lambda _2 +\ldots +(-1)^g\Lambda _{2g}}{\prod 1 - \nu _i\psi _i}, \end{aligned}$$

where *r* denotes the number of simple branch points, the $\psi _i$’s should be closely related to the pull-back of the homonymous classes on $\overline{\mathcal {M}}_{g,n}$ and the $\Lambda _{2i}$ are some yet unidentified classes of degree 2*i*. Expression (11) renders various properties (degree, parity, and vanishing of coefficients in certain degree ranges) of the one-part Hurwitz polynomial transparent.

While the Goulden-Jackson-Vakil conjecture in its strong form (11) is still wide open, some results [5, 8] expressed one-part double Hurwitz numbers as intersection numbers on moduli spaces of curves, confirming the predicted polynomial and integrability structure. Blot [2] established a connection between one-part double Hurwitz numbers and the quantum KdV hierarchy, showing that the coefficients of $H_g((d),\boldsymbol{\nu })$ coincide with the expansion of the quantum Witten–Kontsevich series.

Cavalieri, Markwig, and Ranganathan [6] introduced *k*-leaky double Hurwitz numbers $H_g^k(\boldsymbol{\mu },\boldsymbol{\nu })$, which relax the usual degree-balancing condition by allowing a controlled discrepancy: $\sum \mu _i - \sum \nu _j = k \cdot (2g-2)$. These invariants are defined via logarithmic geometry as intersection numbers against pluricanonical double ramification cycles. They generalize ordinary double Hurwitz numbers (recovered when k=0) and maintain key properties: for fixed *g* and *k*, they are piecewise polynomial in the parts of $\boldsymbol{\mu }$ and $\boldsymbol{\nu }$, with well-defined chamber walls.

Descendant insertions, or ψ-classes, are natural elements of the Chow ring of $\overline{\mathcal {M}}_{g,n}$. The structure of their intersection numbers was unveiled in Witten’s conjecture (Kontsevich’s theorem)[16], stating that a generating function for all intersection numbers of ψ-classes is a τ-function for the KdV hierarchy. Buryak, Shardin, Spitz and Zvonkine [3] describe the structure of intersection numbers of ψ-classes against the double ramification cycle.

Cavalieri, Markwig, and Schmitt [7] defined *k*-leaky double Hurwitz descendants, incorporating ψ-classes at the special ramification points. These descendant invariants also exhibit piecewise polynomiality and satisfy explicit wall-crossing formulas in genus 0.

Recent work by Accadia, Karev, and Lewański [1] approaches *k*-leaky Hurwitz numbers from the bosonic Fock space perspective, extending the basis of symmetric functions to include completed cycle operators. This provides a new proof of piecewise polynomiality in the leaky setting and suggests a potential ELSV-type formula involving completed cycles and quantum intersection numbers.

## Background: Tropical Leaky Covers

To set the notation for tropical leaky covers, we follow the exposition of [7]: An *abstract tropical* *curve* is a connected metric graph Γ whose leaf edges (called *ends*) have infinite length, together with a genus function $g:\Gamma \rightarrow \mathbb {Z}_{\ge 0}$ with finite support. Locally around a point *p*, Γ is homeomorphic to a star with *r* half-edges. The number *r* is called the *valence* of the point *p* and denoted by ${{\,\textrm{val}\,}}(p)$. The *minimal vertex set* of Γ is defined to be the points where the genus function is non-zero, together with points of valence different from 2. Besides *edges*, we introduce the notion of *flags* of Γ. A flag is a pair $(\textrm{v,e})$ of a vertex $\textrm{v}$ and an edge $\textrm{e}$ incident to it ($\textrm{v}\in \partial \textrm{e}$). Edges that are not ends are required to have finite length and are referred to as *bounded* or *internal* edges.

A *marked tropical curve* is a tropical curve whose leaves are labeled. An isomorphism of a tropical curve is an isometry respecting the leaf markings and the genus function. The *genus* of a tropical curve Γ is given by

$$ g(\Gamma ) = h_1(\Gamma )+\sum _{p\in \Gamma } g(p). $$

The *combinatorial type* is the equivalence class of tropical curves obtained by identifying any two tropical curves which differ only by edge lengths.

We study covers of $\mathbb {R}$ by graphs up to additive translation, and equip $\mathbb {R}$ with a polyhedral subdivision to ensure the result is a map of metric graphs (see e.g. Section 5.4 and Figure 3 in [14]). A *metric line graph* is any metric graph obtained from a polyhedral subdivision of $\mathbb {R}$. The metric line graph determines the polyhedral subdivision up to translation. We fix an orientation of a metric line graph going from left to right (i.e. from negative values in $\mathbb {R}$ to positive values).

### Definition 4

(Leaky cover, [6]) Let $\pi :\Gamma \rightarrow T$ be a surjective map of metric graphs where *T* is a metric line graph. We require that π is piecewise integer affine linear, the slope of π on a flag or edge $\textrm{e}$ is a positive integer called the *expansion factor* $\omega (\textrm{e})\in \mathbb {N}_{\ge 1}$.

For a vertex $\textrm{v}\in \Gamma$, the *left (resp. right) degree of *π* at *$\textrm{v}$ is defined as follows. Let $f_l$ be the flag of $\pi (\textrm{v})$ in *T* pointing to the left ($f_r$ the flag pointing to the right). Add the expansion factors of all flags *f* adjacent to *v* that map to $f_l$ (resp. $f_r$):

12 $$\begin{aligned} d_\textrm{v}^l=\sum _{f\mapsto f_l} \omega (f), \;\;\;\; d_\textrm{v}^r=\sum _{f\mapsto f_r} \omega (f). \end{aligned}$$

We say that the *k*-leaky condition is satisfied at $\textrm{v}\in \Gamma$ if13We impose a stability condition: $\Gamma \rightarrow T$ is called stable if the preimage of every vertex of *T* contains a vertex of Γ in its preimage which is of genus greater than 0 or valence greater than 2.

Furthermore, we stabilize the source tropical curve further by passing to its minimal vertex set (containing only the points where the genus function is non-zero, together with points of valence different from 2). The outcome $\pi :\Gamma \rightarrow T$ is called a *k-leaky cover*.

By the stabilization procedure, we lose the property that the cover is a map of graphs, however, this vertex structure is relevant to determine valencies correctly for the purpose of ψ-conditions.

### Example 5

**Fig. 1:**
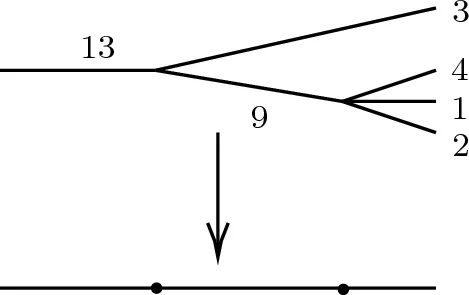
A 1-leaky cover

Figure 1 shows an example of a 1-leaky cover from a tropical curve Γ of genus 0 (i.e. a metric tree, in particular there is no genus hidden at points). We do not specify the metric structure of Γ in the picture, as it is obtained from the metric structure of the line graph (i.e. the length of the bounded edge of Γ is $\frac{1}{9}$ times the distance of the two images of vertices in the line graph.) The source Γ has two vertices. We obtain a map of graphs by introducing a 2-valent vertex in Γ in the edge of weight 3 for the other preimage of the image of the 4-valent vertex.

### Definition 6

*(Left and right degree)* The *left (resp. right) degree* of a leaky cover is the tuple of expansion factors of its ends mapping asymptotically to -∞ (resp. +∞). The tuple is indexed by the labels of the ends mapping to -∞ (resp. +∞). When the order imposed by the labels of the ends plays no role, we drop the information and treat the left and right degree only as a multiset.

For the leaky cover in Example 5, the left degree is 13 and the right degree is (1, 2, 3, 4).

By convention, we denote the left degree by $\textbf{x}^{+}$ and the right degree by $\textbf{x}^{-}$. In the right degree, we use negative signs for the expansion factors, in the left degree positive signs. We also merge the two to one vector which we denote $\textbf{x}=(x_1,\ldots ,x_n)$ called the *degree*. In Example 5, the degree equals (13,-1,-2,-3,-4). The labeling of the ends plays a role: the expansion factor of the end with the label *i* is $x_i$. In $\textbf{x}$, we distinguish the expansion factors of the left ends from those of the right ends by their sign.

We focus on one part leaky covers, i.e. when $\textbf{x}=(d, -\boldsymbol{\nu })$. In this case we let n=m+1 and index the components of $\boldsymbol{\nu }$ from 1 to *m*. An Euler characteristic calculation, combined with the leaky cover condition (13), shows that

$$\begin{aligned} d-\sum _{i=1}^m \nu _i=k\cdot (2\,g-2+n), \end{aligned}$$

where *g* denotes the genus of Γ. An automorphism of a leaky cover is an automorphism $\phi :\Gamma \rightarrow \Gamma$ of the marked tropical curve, preserving the genus function and end labels, such that $\pi \circ \phi =\pi$.

For Example 5, the global balancing condition is $13-(1+2+3+4)=3=1\cdot (2\cdot 0-2+5)$. The two local balancing equations are 13-(3+9)=1 at the left vertex and 9-(4+1+2)=2 at the right vertex.

For a genus-zero one-part leaky cover, root the source tree at the vertex adjacent to the end of weight *d*. If $\textrm{e}$ is a bounded edge and $I_\textrm{e}$ is the set of right ends separated from the root by $\textrm{e}$, then summing the leaky balancing condition over the component away from the root gives

$$ \omega (\textrm{e})=\sum _{i\in I_\textrm{e}}\nu _i+k\cdot (|I_\textrm{e}|-1). $$

Thus, for k≥0 and $\nu _i>0$, all such slopes are positive; in particular, every bounded edge points to the right when oriented away from the root, so the root (the vertex adjacent to the end of weight *d*) is the leftmost vertex of the cover. The corresponding chamber closure is determined by $\sum _{i\in I}\nu _i\ge -k\cdot (|I|-1)$ for every nonempty subset $I\subseteq \{1,\ldots ,m\}$, in accordance with the genus zero wall description of [7]; under our standing assumptions the one-part data lies in its interior.

For the remaining part of the section, let g≥0 such that 2g-2+n>0 and consider vectors $(d, -\boldsymbol{\nu })$ such that $d-\sum _{i=1}^m\nu _i = k \cdot (2g-2+n)$ for some $k \in \mathbb {Z}_{\ge 0}$. Let $\textbf{e}\in \mathbb {Z}_{\ge 0}^n$ such that $0 \le |\textbf{e}| \le 2g-3+n$.

### Definition 7

*(*ψ-*conditions for leaky covers)* Let $\pi :\Gamma \rightarrow T$ be a *k*-leaky cover. For a vertex $\textrm{v}$, let $I_\textrm{v}\subset \{1,\ldots ,n\}$ be the subset of ends adjacent to $\textrm{v}$ after passing to the minimal vertex set of Γ. We say that $\pi :\Gamma \rightarrow T$ *satisfies the *ψ*-conditions* $\textbf{e}$ if for all vertices $\textrm{v}$ of Γ,

14 $$\begin{aligned} \sum _{i\in I_\textrm{v}}e_i =2g(\textrm{v})-3+ {{\,\textrm{val}\,}}(\textrm{v}).\end{aligned}$$

Here, the sum on the left is 0 if $I_\textrm{v}=\emptyset$.

The leaky cover in Example 5 satisfies the ψ-conditions $\textbf{e}=(0,0,0,0,1).$

### Definition 8

*(Vertex multiplicities)* Let $\pi :\Gamma \rightarrow T$ be a *k*-leaky cover satisfying the ψ-conditions $\textbf{e}$. For a vertex $\textrm{v}$, let $I_\textrm{v}\subset \{1,\ldots ,n\}$ be the subset of ends adjacent to $\textrm{v}$ after passing to the minimal vertex set of Γ. Let $\textbf{x}(\textrm{v})$ denote the vector containing the (left and right) local degree of $\textrm{v}$, let $g(\textrm{v})$ denote the genus of $\textrm{v}$.

We define the *vertex multiplicity* to be

$$\begin{aligned} {{\,\textrm{mult}\,}}_\textrm{v}:= \int _{\overline{M}_{g(\textrm{v}),{{\,\textrm{val}\,}}(\textrm{v})}} {{\,\textrm{DR}\,}}_{g(\textrm{v})}(\textbf{x}(\textrm{v}))\cdot \prod _{i\in I_\textrm{v}} \psi _i^{e_i} \end{aligned}$$

### Definition 9

*(Count of* *k*-*leaky covers satisfying* ψ-*conditions)*

We define

15 $$\begin{aligned} \textsf{H}^{{{\,\textrm{trop}\,}}}_g((d, -\boldsymbol{\nu }), \textbf{e}) = \sum _\pi {{\,\textrm{mult}\,}}_\pi , \end{aligned}$$

where:

- $\pi :\Gamma \rightarrow T$ ranges among all leaky covers of degree $(d, -\boldsymbol{\nu })$ and genus *g* (Definition 4) and satisfying the ψ-conditions $\textbf{e}$ (Definition 7); we require that every vertex of *T* has precisely one vertex in its preimage;
- The multiplicity
  16 $$\begin{aligned} {{\,\textrm{mult}\,}}_\pi = \frac{1}{|{{\,\textrm{Aut}\,}}(\pi )|}\cdot \prod _\textrm{e} \omega (\textrm{e})\cdot \prod _\textrm{v} {{\,\textrm{mult}\,}}_\textrm{v} \in \mathbb {Q} \end{aligned}$$
  where the first part is the product of the expansion factors at the bounded edges of Γ (according to its minimal vertex set), weighted by the number of automorphisms of π; the last product goes over the set of vertices of Γ with ${{\,\textrm{mult}\,}}_\textrm{v}$ as in Definition 8. Here, an automorphism of a cover is an automorphism of the marked tropical source, preserving the genus function and end labels, and respecting the covering map. By our convention to label the ends, the only non-trivial automorphisms can come from parallel bounded edges of Γ.

### Remark 10

([7], Remark 5.2) In genus 0, the vertex multiplicity

$$\begin{aligned} {{\,\textrm{mult}\,}}_\textrm{v}= \frac{({{\,\textrm{val}\,}}(\textrm{v})-3)!}{\prod _{i\in I_\textrm{v}}e_i!} \end{aligned}$$

of a leaky cover satisfying ψ-conditions does not depend on *k* or on the expansion factors of its adjacent edges; it equals a multinomial coefficient that only depends on its valence and the ψ-conditions.

The leaky cover in Example 5 has multiplicity 9: it has no non-trivial automorphisms, as we view the ends as labeled. The weight of its only bounded edge is 9. The vertex multiplicities are 1 for both vertices.

### Theorem 11

([7], Theorem 1.2) The *k*-leaky double Hurwitz descendant (9) equals the count of tropical *k*-leaky covers satisfying ψ-conditions (Definition 9):

$$\begin{aligned} \textsf{H}^{{{\,\textrm{trop}\,}}}_g((d, -\boldsymbol{\nu }), \textbf{e})=\textsf{H}_g((d, -\boldsymbol{\nu }), \textbf{e}). \end{aligned}$$

### Remark 12

The hypothesis k≥0 is sufficient, but not necessary, for the validity of our results. In genus zero, our proofs only use that all bounded edges of the contributing tropical covers have positive weight, which by the discussion above holds whenever $\sum _{i\in I}\nu _i > -k\cdot (|I|-1)$ for all nonempty subsets $I\subseteq \{1,\ldots ,m\}$; hence the genus zero results of this paper hold for arbitrary $k\in \mathbb {Z}$, keeping $d, \nu _i > 0$, under these inequalities. The results extend further to the chamber closure, where some of the inequalities $\sum _{i\in I}\nu _i \ge -k\cdot (|I|-1)$ become equalities, provided the tropical sums and the displayed formulas are interpreted as their polynomial extensions: any summand whose combinatorial type contains a bounded edge of weight zero contributes zero, since its multiplicity contains the product of the bounded edge weights, and in formula (3) the linear denominator $-\nu _1-e_1\cdot \frac{k}{2}$ divides the numerator (as one checks by substituting $\nu _1 = -e_1\cdot \frac{k}{2}$), so that its right hand side remains polynomial. In higher genus, the hypothesis k≥0 similarly enters only by ensuring that the last vertex of a contributing cover does not carry the end of weight *d* and that the cut edge weights appearing in the recursion of Lemma 17 are non-negative; we expect an analogous extension of our results to the closure of the corresponding chamber, whose explicit description we do not pursue here.

## Genus Zero

We study the case of rational invariants. Throughout this section, we set g=0 and consider $(d, - \boldsymbol{\nu }) = (d, -\nu _1, \ldots , -\nu _m)$ of length n=m+1, where $d, \nu _1, \ldots , \nu _m > 0$ satisfy

17 $$\begin{aligned} d - \sum _{i=1}^m \nu _i = k\cdot (m-1)\,.\end{aligned}$$

### Recursion and Formulas

We first focus on invariants with at most two descendant insertions. In this case we use the shortened notation

$$\begin{aligned} J_{e_0,e_1}^m=\textsf{H}_0((d,-\nu _1,\ldots ,-\nu _m),(e_0,e_1,0,\ldots ,0)), \end{aligned}$$

where we observe that $J_{e_0,e_1}^m$ is a function of $d,\nu _i,k,e_0,e_1$ and *m*, but we leave visible only the variables that participate in the recursions we use. It is convenient to set $J_{e_0,e_1}^m:= 0$ when the parameters lie outside the allowed range, i.e. when m≤1 or $e_0+e_1 > m-2$; this convention makes the right hand sides of the recursions below well-defined also in the boundary cases (e.g. for m=2 in Lemma 13). The next two lemmas develop recursions among these invariants.

#### Lemma 13

Given m≥2 and $0\le e_0 \le m-2$, we have

18 $$\begin{aligned} J_{e_0,0}^m=(m-1)\cdot \left( d-(m-2)\cdot \frac{k}{2}\right) \cdot J_{e_0,0}^{m-1} +\delta _{m-2, e_0}. \end{aligned}$$

with the base cases of the recursion encoded by the Kronecker delta $\delta _{m-2,e_0}$. In particular the invariants $J_{e_0, 0}^m$ are independent of $\boldsymbol{\nu }$, the negative ramification profile.

#### Proof

We prove the independence of $\boldsymbol{\nu }$ by induction on *m*, with the base case m=2 being true because the invariant $J^2_{0,0} = 1$. This also establishes (18) for m=2. For m>2, since $J^m_{m-2,0} = \int _{\overline{\mathcal {M}}_{0,m+1}}\psi _0^{m-2} = 1$, (18) holds for $e_0 = m-2$.

By Theorem 11, the numbers $J_{e_0, 0}^m$ are calculated as a weighted count of tropical *k*-leaky covers, which for $e_0<m-2$ have at least two vertices. Recall from Sect. 2 that since k≥0, the vertex adjacent to the end of weight *d* is the leftmost vertex of any such cover. In particular, the last (rightmost) vertex cannot carry the end of weight *d*; it therefore supports no ψ-conditions and is 3-valent, so that all cases are covered by the following analysis. To establish the recursive part of (18), we cut each tropical cover just before the last vertex, which has multiplicity 1. If it is adjacent to the ends of weight $\nu _i$ and $\nu _j$, the weight of its adjacent bounded edge is $\nu _i+\nu _j+k$. Cutting this edge, the remainder of the graph (to the left of the cut) contributes to $J_{e_0,0}^{m-1}$. Since all graphs contributing to $J_{e_0,0}^{m-1}$ appear exactly once this way, we have

19 $$\begin{aligned} J_{e_0,0}^m = \sum _{1\le i<j\le m} (\nu _i+\nu _j+k)\cdot J_{e_0,0}^{m-1} =\left( \sum _{1\le i<j\le m} (\nu _i+\nu _j)+\sum _{1\le i<j\le m} k\right) \cdot J_{e_0,0}^{m-1}. \end{aligned}$$

We observe that (19) is unambiguous (and in fact the last term is well-defined) because by inductive hypothesis the invariants $J_{e_0,0}^{m-1}$ are independent of the negative ramification profiles. The first summand in the last term of (19) equals $(m-1)\cdot (\nu _1+\ldots +\nu _m)$, which by (17) then equals $(m-1)\cdot (d-k\cdot (m-1))$. The second summand equals ${{m}\atopwithdelims (){2}}\cdot k$. Thus (19) becomes:

20 $$\begin{aligned} J_{e_0,0}^m = \left( (m-1)\cdot (d-k\cdot (m-1))+{{m}\atopwithdelims (){2}}\cdot k\right) \cdot J_{e_0,0}^{m-1}, \end{aligned}$$

which is easily seen to agree with (18) (since $e_0<m-2$ the Kronecker delta equals zero). Formula (18) immediately establishes the independence of $J^m_{e_0, 0}$ from $\boldsymbol{\nu }$.

□

#### Lemma 14

Given m≥3, $e_0\ge 0$, $e_1>0$ and $e_0+e_1\le m-2$, we have

21 $$\begin{aligned}&J_{e_0,e_1}^m=\nonumber \\  &\Big (\left( {\begin{array}{c}m-1\\ e_1+1\end{array}}\right) \cdot (k \cdot (e_1+1)+\nu _1)+ \left( {\begin{array}{c}m-2\\ e_1\end{array}}\right) \cdot (d-\nu _1-k\cdot (m-1)) \Big )\cdot J_{e_0,0}^{m-e_1-1}\nonumber \\  &+\Big (\left( {\begin{array}{c}m-1\\ 2\end{array}}\right) \cdot k+(m-2)\cdot (d-\nu _1-k\cdot (m-1))\Big )\cdot J_{e_0,e_1}^{m-1} +\left( {\begin{array}{c}m-2\\ e_0\end{array}}\right) \cdot \delta _{m-2,e_0+e_1}\,, \end{aligned}$$

with the base cases of the recursion again encoded by the Kronecker delta part of the formula. In particular the invariants $J_{e_0, e_1}^m$ are independent of $\nu _2, \ldots , \nu _m$, the ramification conditions at points that do not support a positive power of a ψ-class.

#### Proof

The proof is very similar to the proof of Lemma 13, so we move along a bit faster. In particular, we omit weaving the inductive proof of independence from $\nu _2, \ldots , \nu _m$ with the establishing of the recursion, as it goes exactly as before and would only burden the interesting part. The base cases are evaluation of two-term monomials in ψ-classes on moduli spaces of rational stable curves, which are well-known to produce the above binomial coefficients.

**Fig. 2:**
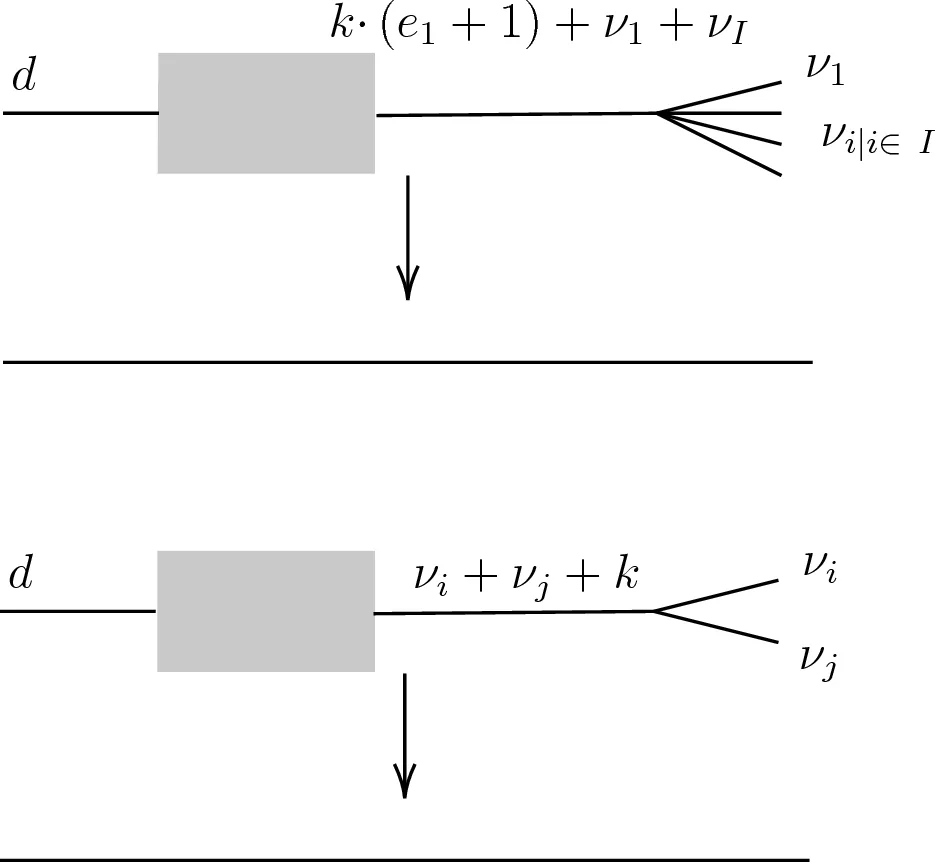
Sketch for the recursion for the invariants $J_{e_0,e_1}(m)$. The tropical leaky covers continue in some way within the grey boxes, we only pay attention to the behavior of the last vertex

Letting $e_0+e_1<m-2$, and applying the same vertex cutting algorithm as in the proof of Lemma 13, we must now consider two cases (see Fig. 2):

**Case 1:** The last vertex is adjacent to the end marked 1 of weight $\nu _1$, in which case it has to be $e_1+3$-valent. As it is adjacent to a single bounded edge, it must be adjacent to $e_1+1$ more ends. By Remark 10, its local vertex multiplicity is one, independently of the weight of the other adjacent ends. Let $I\subset \{2\ldots ,m\}$ be the subset of additional ends adjacent to the last vertex. We use the notation
  $$\begin{aligned} \nu _I:= \sum _{i\in I} \nu _i. \end{aligned}$$
  The bounded edge which we cut is then of weight
  $$\begin{aligned} k \cdot (e_1+1)+\nu _1+\nu _I. \end{aligned}$$
  The part that remains (to the left of the cut) is a tropical leaky cover satisfying a $\psi _0^{e_0}$ condition on the left end of weight *d*, and has $e_1+1$ fewer right ends. It thus contributes to $J_{e_0,0}^{m-e_1-1}$. Vice versa, one can prolong any such cover by re-attaching the last vertex, the multiplicity changes by a factor of $k \cdot (e_1+1)+\nu _1+\nu _I$. Altogether, this first case contributes
  22 $$\begin{aligned} \sum _{\tiny { \begin{array}{c} I\subset \{2,\ldots ,m\} \\ | I| = e_1+1 \end{array} }} \big (k \cdot (e_1+1)+\nu _1+\nu _I\big ) \cdot J_{e_0,0}^{m-e_1-1}. \end{aligned}$$
  To simplify (22), we rewrite it as follows:
  $$\begin{aligned} \sum _{\tiny { \begin{array}{c} I\subset \{2,\ldots ,m\} \\ | I| = e_1+1 \end{array} }} \big (k \cdot (e_1+1)+\nu _1\big ) \cdot J_{e_0,0}^{m-e_1-1}+ \sum _{\tiny { \begin{array}{c} I\subset \{2,\ldots ,m\} \\ | I| = e_1+1 \end{array} }} \nu _I \cdot J_{e_0,0}^{m-e_1-1} . \end{aligned}$$
  The first summation consists of $$\left( {\begin{array}{c}m-1\\ e_1+1\end{array}}\right) $$ terms which do not depend on *I*, and therefore add up to
  23 $$\begin{aligned} \left( {\begin{array}{c}m-1\\ e_1+1\end{array}}\right) \cdot \big (k \cdot (e_1+1)+\nu _1\big )\cdot J_{e_0,0}^{m-e_1-1} \end{aligned}$$
  For the second summation, note that each $\nu _i$ with $2 \le i \le m$ is contained in exactly $$\left( {\begin{array}{c}m-2\\ e_1\end{array}}\right) $$ of the subsets *I*; comparing coefficients of the $\nu _i$ on both sides, we obtain
  24 $$\begin{aligned} \sum _{\tiny { \begin{array}{c} I\subset \{2,\ldots ,m\} \\ | I| = e_1+1 \end{array} }} \nu _I = \left( {\begin{array}{c}m-2\\ e_1\end{array}}\right) \cdot \sum _{i=2}^{m}\nu _i. \end{aligned}$$
  From the *k*-leaky balancing condition (17) we have:
  25 $$\begin{aligned} \sum _{i=2}^m \nu _i = (d-\nu _1-k\cdot (m-1)) \end{aligned}$$
  Combining (24) and (25) one obtains that the second summation equals:
  26 $$\begin{aligned} \left( {\begin{array}{c}m-2\\ e_1\end{array}}\right) \cdot (d-\nu _1-k\cdot (m-1))\cdot J_{e_0,0}^{m-e_1-1}. \end{aligned}$$
  Adding (23), (26) it is immediate to see that (22) agrees with the first line of the recursion in (21).
**Case 2:** If the last vertex is not adjacent to the end of weight $\nu _1$, it must be 3-valent and have multiplicity 1. If it is adjacent to the ends of weight $\nu _i$ and $\nu _j$, where i,j≠1, the weight of its adjacent bounded edge is $\nu _i+\nu _j+k$. Cutting this edge, the remainder of the graph (to the left of the cut) contributes to $J_{e_0,e_1}^{m-1}$. Similarly to before, to any contributing graph one may attach a tripod to undo the cut. Thus, the second case contributes
  27 $$\begin{aligned} \sum _{\{i,j\}\subseteq \{2,\ldots ,m\}} (\nu _i+\nu _j+k)\cdot J_{e_0,e_1}^{m-1}. \end{aligned}$$
  Expression (27) is linear symmetric in $\nu _2, \ldots , \nu _m$. Every $\nu _i$ appears in m-2 terms, thus we obtain
  28 $$\begin{aligned}&\Big (\left( {\begin{array}{c}m-1\\ 2\end{array}}\right) \cdot k+(m-2)\cdot (\sum _{i=2}^m \nu _i)\Big )\cdot J_{e_0,e_1}^{m-1}=\nonumber \\  &\Big (\left( {\begin{array}{c}m-1\\ 2\end{array}}\right) \cdot k+(m-2)\cdot (d-\nu _1-k\cdot (m-1))\Big )\cdot J_{e_0,e_1}^{m-1}. \end{aligned}$$

Adding the contributions (23), (26) and (28) from the two cases, we obtain recursion (21).

□

The recursions from Lemmas 13, 14 are used to prove the formulas from Theorem 1.

***Proof of Theorem ***1 (2)***, case ***$e_1=0$: For $e_1=0$, we use the recursion from Lemma 13. Having already established the base cases, we show that for $e_0<m-2$ the right hand side of equation (2) satisfies the recursion from Lemma 14, then the statement follows.

Inserting $J_{e_0,0}^{m-1}= \frac{(m-2)!}{(e_0+1)!} \prod _{j=e_0+1}^{m-3}(d-j\frac{k}{2})$ into the right hand side of (18), we obtain

$$\begin{aligned}&(m-1)\cdot \Big (d-(m-2)\frac{k}{2}\Big ) \frac{(m-2)!}{(e_0+1)!} \cdot \Big ( d-(e_0+1)\frac{k}{2}\Big )\cdot \ldots \cdot \Big ( d-(m-3)\frac{k}{2}\Big )=\\  &\frac{(m-1)!}{(e_0+1)!} \cdot \Big ( d-(e_0+1)\frac{k}{2}\Big )\cdot \ldots \cdot \Big ( d-(m-2)\frac{k}{2}\Big )=J_{e_0,0}^m\end{aligned}$$

as required.

□

***Proof of Theorem ***1 (3)***, case ***$e_0=0$ The base case is $J^m_{0,e_1} = 1$ when $e_1 = m-2$, which agrees with the right hand side of (3) evaluated at $e_1 = m-2$.

To show that the right hand side of (3) satisfies recursion (21), we introduce temporarily the notation:

29 $$\begin{aligned} \widehat{J}^m_{0,e_1} := {J}^m_{0,e_1}\frac{(e_1+1)!}{(m-2)!} = F^m+G^m+H^m, \end{aligned}$$

where: $$ F^m:= e_1 \cdot \frac{\prod _{j=e_1+1}^{m-1} (d-\nu _1-j\cdot \frac{k}{2}) }{-\nu _1-e_1\cdot \frac{k}{2}}, G^m:= e_1 \cdot \frac{ - \prod _{j=1}^{m-e_1-1} (d-j\cdot \frac{k}{2})}{-\nu _1-e_1\cdot \frac{k}{2}}, H^m:= (m-e_1-1) \cdot \prod _{j=1}^{m-e_1-2} \left( d-j\cdot \frac{k}{2}\right) .$$

Plugging $J_{0,0}^{m-e_1-1} = (m-e_1-2)! \prod _{j = 1}^{m-e_1-2} \left( d-j\frac{k}{2}\right)$ and doing some elementary factorizations, one sees that (21) is equivalent to:

30 $$\begin{aligned} \widehat{J}^m_{0,e_1} = ((d-\nu _1)(e_1+1)+\nu _1(m-1))\cdot \prod _{j = 1}^{m-e_1-2}\left( d-j\cdot \frac{k}{2}\right) + (d-\nu _1-(m-1)\cdot \frac{k}{2})\cdot \widehat{J}^{m-1}_{0,e_1}. \end{aligned}$$

Consider the second summand of (30). It is immediate that:

31 $$\begin{aligned} F^m = \left( d-\nu _1-(m-1)\cdot \frac{k}{2}\right) \cdot F^{m-1}. \end{aligned}$$

By adding and subtracting $e_1\cdot \frac{k}{2}$ one sees:

32 $$\begin{aligned} G^m&= \left( d-\nu _1-(m-1)\cdot \frac{k}{2}\right) \cdot G^{m-1} \nonumber \\&\qquad +\left( \nu _1+ e_1\cdot \frac{k}{2}\right) \cdot G^{m-1} . \end{aligned}$$

One can conclude the proof by showing that

33 $$\begin{aligned} H^m&= ((d-\nu _1)(e_1+1)+\nu _1(m-1))\cdot \prod _{j = 1}^{m-e_1-2}\left( d-j\cdot \frac{k}{2}\right) + (d-\nu _1-(m-1)\cdot \frac{k}{2})\cdot H^{m-1} \nonumber \\&-\left( \nu _1+ e_1\cdot \frac{k}{2}\right) \cdot G^{m-1}, \end{aligned}$$

since (30) is obtained by adding (31), (32), (33). Factoring out $\prod _{j = 1}^{m-e_1-2}\left( d-j\cdot \frac{k}{2}\right)$ from all terms, (33) reduces to a quadratic expression which is easily verified. □

### Polynomiality

We turn our attention to general genus zero invariants. Their polynomiality on the chamber containing the one-part data follows from the piecewise polynomiality and the genus zero wall description of [7] (see also the end of Sect. 2). While we can’t produce explicit general formulas, we give an independent, recursive proof of this polynomiality, which moreover shows that the invariants only depend on *d*, the leaking *k*, and the ramification orders at the markings supporting descendant insertions.

#### Lemma 15

Fix $\textbf{e}=(e_0, \ldots , e_m) \in \mathbb {Z}^n$ with $e_0\ge 0$, $e_1>0,\ldots ,e_u>0$ and $e_{u+1}=\ldots =e_{m}=0$, let $c=m-2-e_0-\ldots -e_u>0$. We have

34 $$\begin{aligned} \textsf{H}_0((d, - \boldsymbol{\nu }), \textbf{e}) = \sum _{(I,J)} F_{I,J} \cdot \left( {\begin{array}{c}e_I\\ e_{i_1},\ldots ,e_{i_{|I|}}\end{array}}\right) \cdot (\nu _I+\nu _J+(e_I+1)\cdot k) \end{aligned}$$

where $I\subset \{1,\ldots ,u\}$, $J\subset \{u+1\ldots ,m\}$ such that $|I|+|J| = \sum _{i\in I} e_i+2$, $e_I = \sum _{i\in I} e_i$ (similarly for $\nu _I,\nu _J$) and

35 $$\begin{aligned} F_{I,J} = \int _{{{\,\textrm{logDR}\,}}_0(\boldsymbol{\nu }\setminus \{\nu _l\;|\;l\in I\cup J\}) \cup \{\nu _I+\nu _J+(e_I+1)\cdot k\})} \prod _{j\in \{1,\ldots ,u\}\setminus I} \psi _j^{e_j}\cdot {{\,\textrm{branch}\,}}_{c-1}. \end{aligned}$$

#### Proof

We obtain this recursion by cutting all tropical leaky covers which contribute to $\textsf{H}_0((d, - \boldsymbol{\nu }), \textbf{e})$ right before their last vertex. The right pointing ends attached to such a vertex identify a subset *I* of $\{1, \ldots , u\}$ and a subset *J* of $\{u+1, \ldots , m\}$ such that $|I|+|J| = \sum _{i\in I} e_i+2$, and vice-versa for any pair of subsets we can find graphs with a vertex of that type, which we call $V_{I,J}$. The unique left pointing end of $V_{I,J}$ has weight $(\nu _I+\nu _J+(e_I+1)\cdot k)$, where $\nu _I = \sum _{i\in I} \nu _i$ and similarly for $\nu _J$. Finally, consider all the graphs that contain a vertex of type $V_{I,J}$: the remaining parts of the graphs to the left of the cut are precisely the leaky tropical covers contributing to (35), thus concluding the proof of the lemma. □

#### Proposition 16

With notation as above, the invariant

$$ \textsf{H}_0((d, - \boldsymbol{\nu }), \textbf{e}) = \int _{{{\,\textrm{logDR}\,}}_0(d, -\boldsymbol{\nu })} \psi _0^{e_0}\psi _1^{e_1}\ldots \psi _u^{e_u}\cdot {{\,\textrm{branch}\,}}_{c}\, $$

is a polynomial of degree *c* in *d*, *k*, and $\nu _1,\ldots ,\nu _u$ for $k\in \mathbb {Z}_{\ge 0}$. In particular, it is independent of the variables $\nu _{u+1}, \ldots , \nu _{m}$ associated to markings without ψ-insertions.

#### Proof

We prove this by induction on $c=m-2-e_0-\ldots -e_u$. If c=0, the integral above reduces to the known integral

$$ \int _{\overline{\mathcal {M}}_{0,m+1}} \psi _0^{e_0} \cdots \psi _u^{e_u} = \left( {\begin{array}{c}m-2\\ e_0, e_1, \ldots , e_u\end{array}}\right) \,, $$

which is constant in *d*, *k* and the entries of $\mathbf {\nu }$. On the tropical side, this corresponds to a single *k*-leaky cover with just one vertex and thus contributing its multiplicity.

Now consider the case c>0. By Lemma 15, $\textsf{H}_0(d, - \boldsymbol{\nu })$ may be written as a finite sum, where the indexing set (*I*, *J*) of the summation does not depend on *d*, *k* or any $\nu _i$. Each summand is the product of three terms: the first, $F_{I,J}$ is an invariant with branch degree equal to c-1, therefore by the inductive hypothesis it is a polynomial of degree c-1 in (a subset of) the variables *d*, *k* and $\nu _1, \ldots , \nu _u$: note that the end of weight $(\nu _I+\nu _J+(e_I+1)k)$ does not support any descendant insertion, and therefore the invariant does not depend on this weight. The second factor is a multinomial coefficient depending only on the descendant vector $\textbf{e}$, therefore it is constant in our variable of interest. The last factor is linear in *k* and all the $\nu _i$’s, so we can already see that $\textsf{H}_0(d, - \boldsymbol{\nu })$ is a polynomial of degree *c* in *d*, *k* and the end weights. We must now show that we can eliminate the dependence on the $\nu _j$ with j∈J. We observe that by the inductive hypothesis $F_{I,J}$ only depends on the subset *I* and not on *J*, so we write $F_I = F_{I,J}$ for any valid choice of *J*. We can hence rewrite (34) as

36 $$\begin{aligned} \textsf{H}_0((d, - \boldsymbol{\nu }), \textbf{e}) = \sum _{I} F_I \cdot \left( {\begin{array}{c}e_I\\ e_{i_1},\ldots ,e_{i_{|I|}}\end{array}}\right) \cdot \sum _{J} \cdot (\nu _I+\nu _J+(e_I+1)\cdot k)\,, \end{aligned}$$

and we focus on the second summation. The summand $\nu _I+(e_I+1)\cdot k$ is the same for each choice of *J*, so in total we obtain it $$ \left( {\begin{array}{c}m-u\\ e_I+2-|I|\end{array}}\right) $$ times. For every $u+1\le j\ \le m$, $\nu _j$ appears in the summation $$\left( {\begin{array}{c}m-u-1\\ e_I+1-|I|\end{array}}\right) $$ times, so we obtain the sum

$$\begin{aligned} \nu _{u+1}+\ldots +\nu _m = d-(m-1)k-\nu _1-\ldots -\nu _u \end{aligned}$$

multiplied with this binomial coefficient. Substituting in (36), we obtain

$$\begin{aligned} \textsf{H}_0((d, - \boldsymbol{\nu }), \textbf{e})&= \sum _{I} F_I \cdot \left( {\begin{array}{c}e_I\\ e_{i_1},\ldots ,e_{i_{|I|}}\end{array}}\right) \cdot \Bigg (\left( {\begin{array}{c}m-u\\ e_I+2-|I|\end{array}}\right) \cdot (\nu _I+(e_I+1)k) \;\;+\\&\qquad \left( {\begin{array}{c}m-u-1\\ e_I+1-|I|\end{array}}\right) \cdot (d-(m-1)k-\nu _1-\ldots -\nu _u)\Bigg ), \end{aligned}$$

which is a polynomial of degree *c* in $d, k, \nu _1, \ldots ,\nu _u$. □

## Higher Genus

In this section we turn our attention to higher genus invariants. In this case we still obtain a recursion by excising the last vertex of tropical covers, but the presence of positive genus allows for more possible terms to the recursion. We then provide some formulas in genus one, and some in arbitrary genus for k=0.

### Recursion for One-Part Leaky Double Hurwitz Numbers

We formulate a recursion for leaky covers which allows for arbitrary genus, but restricted to the case $\textbf{e}=\textbf{0}.$

#### Lemma 17

For genus g≥0, m≥1, and $k\in \mathbb {Z}_{\ge 0}$, the one-part *k*-leaky double Hurwitz numbers $\textsf{H}_g(d, \boldsymbol{\nu }):=\textsf{H}_g((d, -\boldsymbol{\nu }),\textbf{0})$ satisfy the following recursion:

37 $$\begin{aligned} \textsf{H}_g(d, \boldsymbol{\nu })&= \sum _{1\le i<j\le m} (\nu _i+\nu _j+k) \cdot \textsf{H}_g(d, \boldsymbol{\nu }\setminus \{\nu _i,\nu _j\} \cup \{\nu _i+\nu _j+k\}) \end{aligned}$$

38 $$\begin{aligned}&+ \frac{1}{2} \sum _{i=1}^m \sum _{\begin{array}{c} a+b= \nu _i+k\\ a,b\ge 0 \end{array}} a \cdot b \cdot \textsf{H}_{g-1}(d, \boldsymbol{\nu }\setminus \{\nu _i\} \cup \{a, b\}) \end{aligned}$$

39 $$\begin{aligned}&- \frac{k}{24} \cdot \textsf{H}_{g-1}(d, \boldsymbol{\nu }\cup \{k\}) \end{aligned}$$

40 $$\begin{aligned}&+ \frac{1}{6} \sum _{\begin{array}{c} a+b+c=k\\ a,b,c\ge 0 \end{array}} a \cdot b \cdot c \cdot \textsf{H}_{g-2}(d, \boldsymbol{\nu }\cup \{a,b,c\}), \end{aligned}$$

where terms with negative genus are omitted (equivalently, interpreted as zero). We have as base cases:

41 $$\begin{aligned} \textsf{H}_0(d, \boldsymbol{\nu })&= (m-1)! \cdot \prod _{j=1}^{m-2} \left( d - j \cdot \frac{k}{2}\right) \end{aligned}$$

The assumption k≥0 ensures that the edge weights appearing in the cut are non-negative: the first term has weight $\nu _i+\nu _j+k$, the second sums over non-negative splittings of $\nu _i+k$, the third introduces an end of weight *k*, and the fourth sums over non-negative triples with total weight *k*. Thus the nonzero terms in the recursion only use positive expansion factors.

**Fig. 3:**
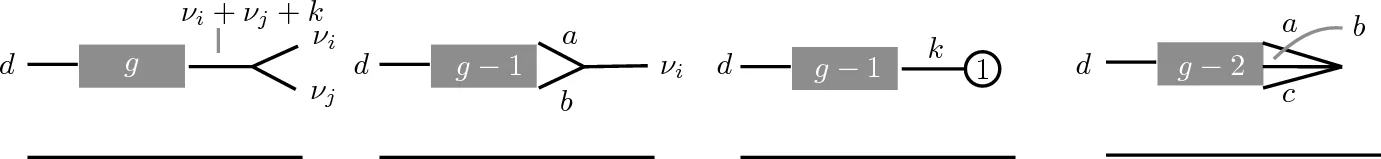
The four possible local structures of leaky covers at the last vertex

#### Proof

The proof is analogous to the recursion in Lemma 14. We cut the last vertex of a *k*-leaky tropical cover, compute the contribution of this cut part, and notice that the remaining part of the cover contributes to an invariant where the quantity (2g-1+m) has decreased by one. The four terms in this recursion correspond to different local structures at the last vertex of a tropical *k*-leaky cover, as illustrated in Fig. 3:

1. The last vertex carries two markings $\nu _i$ and $\nu _j$, connected to the rest of the curve by one bounded edge.
2. The last vertex carries one marking $\nu _i$, connected to the rest of the curve by two bounded edges, which lowers the genus of the remaining part by 1.
3. The last vertex forms a cul-de-sac (a one-valent genus one vertex with one bounded edge and no markings), which lowers the genus by 1.
4. The last vertex has three bounded edges connecting to the rest of the curve, which lowers the genus by 2.

In this formula, *k* is determined by the balancing condition $d-\sum _{i=1}^m \nu _i = k \cdot (2g+m-1)$. In the first case, the last vertex has multiplicity one and the bounded edge which we cut has weight $\nu _i+\nu _j+k$. The remaining cover after cutting this bounded edge still has genus *g*. Instead of the two right ends of weights $\nu _i,\nu _j$ it has one right end of weight $\nu _i+\nu _j+k$.

In the second case, the last vertex is still 3-valent, but we cut two bounded edges. If the vertex carries the marking $\nu _i$ and we set the weight of one of these edges to be *a*, then by the leaky balancing condition, the second has weight $b = \nu _i+k-a$. Summing over all *i* and all *a*, *b* such that $a+b = \nu _i+k$ we double count isomorphic leaky covers that appear. For that reason, we multiply with a factor of $\frac{1}{2}$.

In the third case, the vertex we cut off has a local multiplicity of $-\frac{1}{24}$ (see e.g. Example 6.1 [7]). The genus of the remaining cover is g-1, and it has a new end, of weight *k*.

For the fourth case, the local vertex multiplicity is one. We cut three edges whose weights we can denote by *a*, *b*, and, by leaky balancing, c=k-a-b. The factor of $\frac{1}{6}$ is again obtained by the $S_3$ symmetry in the roles of the three edges, which leads to overcounting covers by a factor of 6 when summing over all *a*, *b*, *c* such that a+b+c=k. □

The recursion from Lemma 17 allows us to generalize the result from [11, Corollary 3.3] on genus one one-part double Hurwitz numbers to arbitrary non-negative *k* and prove Theorem 2.

#### Proof

We apply the recursion from Lemma 17 in the case g=1, inserting the formula from (6) and the base case (41); the right hand side of the recursion is:

$$\begin{aligned}\hspace{-1cm}&\sum _{\{i,i'\}\subseteq \{1,\ldots ,m\}} (\nu _i+\nu _{i'}+k)\cdot \frac{m!}{24}\cdot \prod _{j=2}^{m-1} \left( d - j\cdot \frac{k}{2}\right) \cdot \left[ (d-k)^3 - \left( d-\frac{k}{2}\right) \right. \\  &\left. \cdot \left( 1+ 2\cdot \sum _{s\not \in \{i,i'\}}(\nu _i+\nu _{i'}+2k)\cdot (\nu _s+k) +2\cdot \sum _{\tiny {\begin{array}{c} \{s,t\}\cap \{i,i'\} = \phi \end{array}}}(\nu _s+k)\cdot (\nu _t+k) \right) \right] \\  &+ \frac{1}{12}\cdot \left( \sum _{i= 1}^m ((\nu _i+k)^3-(\nu _i+k))-\frac{k}{2}\right) \cdot m!\cdot \prod _{j=1}^{m-1} \left( d - j\cdot \frac{k}{2}\right) \,, \end{aligned}$$

where we used that

$$ \frac{1}{2} \sum _{a + b = \nu _i + k} a \cdot b = \frac{1}{12}((\nu _i+k)^3 - (\nu _i+k))\,. $$

We factor $\frac{m!}{24}\cdot \prod _{j=2}^{m-1} \left( d - j\cdot \frac{k}{2}\right)$ from the recursion above, and make the substitution $x_i = \nu _i+k$, to obtain

42 $$\begin{aligned}&\sum _{\{i,i'\}\subseteq \{1,\ldots ,m\}}(x_i+x_{i'}-k) \left[ (d-k)^3-\left( d-\frac{k}{2}\right) \right. \nonumber \\  &\left. \cdot \left( 1+2\cdot \sum _{s\not \in \{i,i'\}} (x_i+x_{i'})x_s+ 2\cdot \sum _{\tiny {\begin{array}{c} \{s,t\}\cap \{i,i'\} = \phi \end{array}}} x_sx_t\right) \right] \nonumber \\  &+ 2\cdot \left( (d-k)^3 -3(d-k)\cdot \sigma _2+3\sigma _3-(d-k)-\frac{k}{2}\right) \cdot \left( d - \frac{k}{2}\right) , \end{aligned}$$

where we have manipulated the third line by expressing the sum of cubes in the elementary symmetric functions in the $x_i$’s

$$ \sum _{i=1}^m x_i^3 = \sigma _1^3 - 3\sigma _1\sigma _2+3\sigma _3 $$

and using the relation $\sigma _1 = \sum _{i=1}^m x_i = d-k$.

We must prove that (42) equals formula (6) after factoring out $\frac{m!}{24}\cdot \prod _{j=2}^{m-1} \left( d - j\cdot \frac{k}{2}\right)$; adopting the symmetric function notation this is:

43 $$\begin{aligned} (m+1)\cdot \left( d - m\cdot \frac{k}{2}\right) \cdot \left[ (d-k)^3 - \left( d - \frac{k}{2}\right) \cdot \left( 1+2\sigma _2 \right) \right] . \end{aligned}$$

Both (42) and (43) are polynomials in *d*, *k* and the $x_i$’s with a homogeneous term of degree 4 and one of degree 2. We begin by comparing the degree 2 terms, and observe that they share a common factor of $\left( d - \frac{k}{2}\right)$. The remaining linear part in (42) is

44 $$\begin{aligned}&-\sum _{\{i,i'\}\subseteq \{1,\ldots ,m\}}(x_i+x_{i'}-k) - 2d +k \nonumber \\&= - (m-1)\cdot (d-k) +{{m}\atopwithdelims (){2}}\cdot k -2d+k \nonumber \\&= -(m+1)\cdot \left( d - m\cdot \frac{k}{2}\right) , \end{aligned}$$

also agreeing with the remaining linear part in (43).

Next we observe there are parts of the degree 4 terms which are divisible by $(d-k)^3$. Factoring this term out, in (42) we have:

45 $$\begin{aligned}&\sum _{\{i,i'\}\subseteq \{1,\ldots ,m\}}(x_i+x_{i'}-k) +2d -k \nonumber \\&= -(m+1)\cdot \left( d - m\cdot \frac{k}{2}\right) , \end{aligned}$$

which agrees with the corresponding part in (43).

Factoring out $2\cdot \left( d - \frac{k}{2}\right)$ from all remaining terms, it remains to show that

46 $$\begin{aligned} -\sum _{i\not =i'\in \{1,\ldots ,m\}}(x_i+x_{i'}-k) \cdot \left( \sum _{s\not \in \{i,i'\}} (x_i+x_{i'})x_s+\sum _{\tiny {\begin{array}{c} \{s,t\}\cap \{i,i'\} = \phi \end{array}}} x_sx_t\right) \nonumber \\ -3\cdot (d-k)\cdot \sigma _2+3\sigma _3 = - (m+1)\cdot \left( d - m\cdot \frac{k}{2}\right) \cdot \sigma _2. \end{aligned}$$

The first line on (46) is a symmetric polynomial in the $x_i$’s, which we manipulate to express as polynomial in the elementary symmetric functions $\sigma _1, \sigma _2, \sigma _3$. For the summands that are a multiple of *k*, we have

47 $$\begin{aligned} k\cdot \sum _{\{i,i'\}\subseteq \{1,\ldots ,m\}} \left( \sum _{s\not \in \{i,i'\}} (x_i+x_{i'})x_s+\sum _{\tiny {\begin{array}{c} \{s,t\}\cap \{i,i'\} = \phi \end{array}}} x_sx_t\right) = k\cdot \frac{(m-2)\cdot (m+1)}{2}\cdot \sigma _2, \end{aligned}$$

since there are 2·(m-2) ways for a monomial of the form $x_ax_b$ to appear when *a* or *b* are in $\{i,i'\}$ and ${{m-2}\atopwithdelims (){2}}$ ways when $\{a,b\}\cap \{i,i'\} = \phi$.

For the remaining terms, we have:

48 $$\begin{aligned}&-\sum _{\{i,i'\}\subseteq \{1,\ldots ,m\}}\cdot \left( \sum _{s\not \in \{i,i'\}} (x_i+x_{i'})^2x_s+\sum _{\tiny {\begin{array}{c} \{s,t\}\cap \{i,i'\} = \phi \end{array}}} (x_i+x_{i'}) x_sx_t\right) \nonumber \\&=- (m-2 )\cdot \sum _{a,b = 1}^m x_a^2x_b - \left( 2\cdot 3+ 3\cdot (m-3) \right) \cdot \sigma _3 \nonumber \\&=- (m-2)\cdot \sigma _1\sigma _2 + 3\cdot ((m-2)-2-(m-3))\cdot \sigma _3 \nonumber \\&= -(m-2)\cdot \sigma _1\sigma _2 - 3\cdot \sigma _3, \end{aligned}$$

where from the second to the third line we have applied the identity:

$$ \sum _{a,b = 1}^m x_a^2x_b = \sigma _1\sigma _2 - 3 \sigma _3. $$

Substituting $\sigma _1 = d-k$ and the results of (47), (48) in the left hand side of (46) we have:

49 $$\begin{aligned}&-(m-2)\cdot (d-k)\cdot \sigma _2 - 3\cdot \sigma _3 + k\cdot \frac{(m-2)\cdot (m+1)}{2}\cdot \sigma _2 -3\cdot (d-k)\cdot \sigma _2+3\sigma _3 \nonumber \\  &= -(m+1)\cdot d\cdot \sigma _2 +\frac{m \cdot (m+1)}{2}\cdot k\cdot \sigma _2 \nonumber \\&= - (m+1)\cdot \left( d - m\cdot \frac{k}{2}\right) \cdot \sigma _2, \end{aligned}$$

which agrees with the right hand side of (46), thus concluding the proof. □

### Proof of Theorem 3

In this section we prove Theorem 3 by a different type of recursive structure, obtained by exchanging a ψ-class with a boundary divisor. We specialize to the case k=0, and briefly recall the required constructions. Refer to [3, 5] for background and proofs.

Observe the tautological diagram50where the maps *S*, *s* are source morphisms, and the maps *Br*, *br* are branch morphisms. In the specific case of k=0 the target of the branch morphisms is a symmetric stack quotient of a Losev-Manin space. We denote by 0,∞ the two heavy (and distinguished) marked points in such a space. The 2g-1+m light points are considered unmarked. We denote by $\hat{\psi }_0\in A^1([LM_{2g-1+m}/S^{2g-1+m}])$ the ψ-class at the marked point 0, by $\tilde{\psi }_0$ the ψ-class on ${{\,\textrm{DR}\,}}_g(d, -\boldsymbol{\nu })$ at the unique inverse image of 0, and by $\psi _0$ the ψ-class on $\overline{\mathcal {M}}_{g, m+1}$ at the corresponding mark.

We have the following facts:

- By definition, the branch class in the logarithmic tautological ring of ${{\,\textrm{logDR}\,}}_g(d, -\boldsymbol{\nu })$ is pulled back from Losev-Manin; denoting by $\Delta ^c$ the sum of all boundary strata of codimension *c* we have
  51 $$\begin{aligned} {{\,\textrm{branch}\,}}_c = Br^*([\Delta ^c]); \end{aligned}$$
- Since when k=0 we have Br=br∘c, by projection formula with respect to the morphism *c*,
  52 $$\begin{aligned} \textsf{H}_g((d, - \boldsymbol{\nu }), {\textbf {e}})_{|k = 0}&:= \int _{{{\,\textrm{logDR}\,}}_g(d, -\boldsymbol{\nu })}\mathbf {\psi ^e}{{\,\textrm{branch}\,}}_{2g-2+m-|\textbf{e}|} \nonumber \\&\qquad = \int _{{{\,\textrm{DR}\,}}_g(d, -\boldsymbol{\nu })}\mathbf {\psi ^e}br^*([\Delta ^{2g-2+m-|\textbf{e}|}]); \end{aligned}$$
- The relation between $\hat{\psi }_0$ and $\tilde{\psi }_0$ is given by a slight generalization of Ionel lemma, which is described in this context in [3, Proposition 2.5], [5, Lemma 4.2]:
  53 $$\begin{aligned} d\cdot \tilde{\psi }_0 = br^*(\hat{\psi _0}); \end{aligned}$$
- The relation between $\psi _0$ and $\tilde{\psi }_0$, given in [3, Lemma 2.6] or [5, Lemma 4.3], simplifies because of the full ramification condition *d*:
  54 $$\begin{aligned} \tilde{\psi }_0 = s^*({\psi _0}); \end{aligned}$$
- Denote by $\Delta _{r_\infty }$ the irreducible boundary divisor in $[LM_{r}/S^{r}]$ parameterizing curves where $r_\infty$ light points lie on the same component as the point ∞. The class $\hat{\psi }_0$ admits the following boundary expression [3, Equation (2)]:
  55 $$\begin{aligned} \hat{\psi }_0 = \sum _{r_\infty =1}^{r-1}\frac{r_\infty }{r} [\Delta _{r_\infty }]. \end{aligned}$$

The proof of Theorem 3 proceeds by induction on $e_0$, trading at each step one ψ-class for a boundary divisor. We first prove independently the special case of the maximal power $e_0 = 2g-2+m$ of Theorem 3, in the form of the one-step reduction of Lemma 18: its reformulation in terms of weighted sums of leaky covers (Lemma 19 below) is exactly the input needed in the induction step of the general proof.

#### Lemma 18

For g,m≥0 with 2g-3+m≥0, we have

56 $$\begin{aligned}&\textsf{H}_g((d,-\boldsymbol{\nu }), (2g-2+m, 0, \ldots , 0))|_{k=0} \nonumber \\&\qquad = \frac{d^{-1}}{2g-1+m}\cdot \textsf{H}_g((d,-\boldsymbol{\nu }), ( 2g-3+m, 0, \ldots , 0))|_{k=0}. \end{aligned}$$

#### Proof

We are in the case r=2g-1+m. By dimension reasons and the natural properties of restriction of ψ-classes to boundary strata, we have that for $r_\infty \ge 2$

57 $$\begin{aligned} \hat{\psi }_0^{2g-3+m}\cdot [\Delta _{r_\infty }] = 0. \end{aligned}$$

With this relation and all the ingredients developed before, we now have:

58 $$\begin{aligned}&\textsf{H}_g((d,-\boldsymbol{\nu }), (2g-2+m, 0, \ldots , 0))|_{k=0} \nonumber \\ =&\int _{{{\,\textrm{DR}\,}}_g(d, -\boldsymbol{\nu })}\psi _0^{2g-2+m} \nonumber \\ {\mathop {=}\limits ^{(53), (54)}}&\int _{{{\,\textrm{DR}\,}}_g(d, -\boldsymbol{\nu })}\frac{1}{d^{2g-2+m}}\cdot br^*(\hat{\psi }_0^{2g-2+m}) \nonumber \\ {\mathop {=}\limits ^{\hbox {\tiny {proj.form.}}}}&\int _{[LM_{2g-1+m}/S^{2g-1+m}]}\frac{1}{d^{2g-2+m}}\cdot \hat{\psi }_0^{2g-2+m} \nonumber \\ {\mathop {=}\limits ^{(55)}}&\int _{[LM_{2g-1+m}/S^{2g-1+m}]}\frac{1}{d^{2g-2+m}}\cdot \hat{\psi }_0^{2g-3+m}\cdot \left( \sum _{r_\infty =1}^{2g-2+m}\frac{r_\infty }{2g-1+m}\cdot [\Delta _{r_\infty }] \right) \nonumber \\ {\mathop {=}\limits ^{(57)}}&\int _{[LM_{2g-1+m}/S^{2g-1+m}]}\frac{1}{d^{2g-3+m}}\cdot \hat{\psi }_0^{2g-3+m}\cdot \frac{1}{d}\cdot \frac{1}{2g-1+m} \cdot [\Delta ^1] \nonumber \\ {\mathop {=}\limits ^{(53), (54), (51)}}&\frac{d^{-1}}{2g-1+m} \cdot \int _{{{\,\textrm{DR}\,}}_g(d, -\boldsymbol{\nu })}\psi _0^{2g-3+m}{{\,\textrm{branch}\,}}_1 \nonumber \\ =&\frac{d^{-1}}{2g-1+m} \cdot \textsf{H}_g((d,-\boldsymbol{\nu }), (2g-3+m, 0, \ldots , 0))|_{k=0}. \end{aligned}$$

□

#### Lemma 19

Assume 2g-3+m≥0 and let π range over the leaky covers contributing to $\textsf{H}_g((d,-\boldsymbol{\nu }), (2g-3+m, 0, \ldots , 0))|_{k=0}$, which have exactly two vertices. Denoting by $\textrm{v}_0, \textrm{v}_1$ the first and second vertex of π and by E(π) the set of compact edges between them,^1^ we have:

59 $$\begin{aligned}&\textsf{H}_g((d,-\boldsymbol{\nu }), (2g-2+m, 0, \ldots , 0))|_{k=0} \nonumber \\&\qquad = \frac{d^{-1}}{2g-1+m}\cdot \sum _{\pi } \frac{1}{|{{\,\textrm{Aut}\,}}(\pi )|}\cdot \prod _{i= 0,1}{{\,\textrm{mult}\,}}_{\textrm{v}_i}\cdot \prod _{\textrm{e}\in E(\pi )}w(\textrm{e}). \end{aligned}$$

#### Proof

Since the branch degree of $\textsf{H}_g((d,-\boldsymbol{\nu }), (2g-3+m, 0, \ldots , 0))|_{k=0}$ equals one, summing the ψ-conditions (14) over all vertices shows that the contributing covers indeed have exactly two vertices. The claimed equality is then the interpretation of Lemma 18 in terms of weighted sums of leaky covers, via Theorem 11: the left hand side of Lemma 18 consists of a single graph with a single vertex counted with multiplicity equal to its local vertex multiplicity. The right hand side is a sum over graphs with two vertices, the second being either a trivalent rational vertex or a genus one cul-de-sac (the latter contributing zero, since its adjacent edge has weight k=0). □

We are now ready to tackle the proof of Theorem 3.

#### Proof of Theorem 3

We prove the theorem by induction on $e_0$, with the base case being trivially true.

Denote by $$\Lambda _e$$ the set of all leaky covers contributing to $$\textsf{H}_g((d,-\boldsymbol{\nu }), (e, 0, \ldots , 0))|_{k=0}$$. For $$\pi :\Gamma \rightarrow \mathbb {R} \in \Lambda _e$$ denote by $$\textrm{v}_0$$ the first vertex of the graph. For $$\pi ':\Gamma '\rightarrow \mathbb {R}\in \Lambda _{e-1}$$ denote by $$\mathrm {v'}_0$$ the first vertex, $$\mathrm {v'}_1$$ the second vertex, and by $E^{\prime }$ the set of edges between them. We denote by $${{\,\textrm{Aut}\,}}(E')$$ the set of automorphisms of the multiset $$\{w(\mathrm {e'})\}_{\mathrm {e'}\in E'}$$

For a leaky cover $\pi \in \Lambda _e$, let

$$\begin{aligned} {{\,\textrm{mult}\,}}_\pi ={{\,\textrm{mult}\,}}_{\textrm{v}_0}\cdot {R_\pi }, \end{aligned}$$

and for $\pi '\in \Lambda _{e-1}$, let

$$\begin{aligned} {{\,\textrm{mult}\,}}_{\pi '} =\prod _{i= 0,1}{{\,\textrm{mult}\,}}_{\textrm{v}_i}\cdot \frac{\prod _{\mathrm {e'}\in E'} w(\mathrm {e'})}{|{{\,\textrm{Aut}\,}}(E')|} \cdot {R_{\pi '}}, \end{aligned}$$

where $R_\star$ is a symbol defined by the two equations to denote the “remaining” part of the multiplicity of the leaky cover after factoring out the bit that we want to focus on.

We define a natural function

60 $$\begin{aligned} \varphi :\Lambda _{e-1}\rightarrow \Lambda _e \end{aligned}$$

which, for a graph $\Gamma^{\prime }$ of a leaky cover $$\pi '\in \Lambda _{e-1}$$, contracts the edges in $E^{\prime }$.

For any fixed $\pi :\Gamma \rightarrow \mathbb {R} \in \Lambda _e$, we make the following observations:

1. For any $$\pi '\in \varphi ^{-1}(\pi )$$,
  61 $$\begin{aligned} R_{\pi '} = R_\pi . \end{aligned}$$
2. Let us cut Γ after $$\mathrm {v_0}$$ and, for any $$\pi '\in \varphi ^{-1}(\pi )$$, cut $\Gamma^{\prime }$ after $$\mathrm {v_1}'$$, and consider the fragment of graph containing $$\mathrm {v_0}'$$ with the multiplicity $${{\,\textrm{mult}\,}}_{\pi '}/R_{\pi '}$$. One can see that the weighted sum of these fragments corresponds to exchanging with boundary divisors one of the ψ-classes in the integral (without any branch class) evaluating $${{\,\textrm{mult}\,}}_{\mathrm {v_0}}$$ as in Lemma 19. The vertex $$\mathrm {v_0}$$ corresponds to some genus $$g_{\mathrm {v_0}}$$ and some number of right ends $$m_\mathrm {v_0}$$, such that the quantity $$2g_\mathrm {v_0}-2+m_\mathrm {v_0} = e$$. We therefore have:
  62 $$\begin{aligned} {{\,\textrm{mult}\,}}_{\mathrm {v_0}} = \frac{d^{-1}}{e+1}\cdot \sum _{\pi '\in \varphi ^{-1}(\pi )} \frac{{{\,\textrm{mult}\,}}_{\pi '}}{R_{\pi '}}. \end{aligned}$$
  We are now ready to conclude the proof:
  63 $$\begin{aligned} \textsf{H}_g((d,-\boldsymbol{\nu }), (e, 0, \ldots , 0))|_{k=0}&= \sum _{\pi \in \Lambda _e} {{\,\textrm{mult}\,}}_{\mathrm {v_0}} \cdot R_\pi \nonumber \\&{\mathop {=}\limits ^{(62)}} \frac{d^{-1}}{e+1}\cdot \sum _{\pi \in \Lambda _e}\sum _{\pi '\in \varphi ^{-1}(\pi )} \frac{{{\,\textrm{mult}\,}}_{\pi '}}{R_{\pi '}} \cdot R_\pi \nonumber \\&{\mathop {=}\limits ^{(61)}} \frac{d^{-1}}{e+1}\cdot \sum _{\pi '\in \Lambda _{e-1}} {{\,\textrm{mult}\,}}_{\pi '} \nonumber \\&= \frac{d^{-1}}{e+1}\cdot \textsf{H}_g((d,-\boldsymbol{\nu }), ( e-1, 0, \ldots , 0))|_{k=0} \nonumber \\&= \frac{d^{-e}}{(e+1)!}\cdot \textsf{H}_g((d,-\boldsymbol{\nu }), ( 0, \ldots , 0))|_{k=0}, \end{aligned}$$
  where the last line is obtained by plugging in the inductive hypothesis, thus concluding the proof.

□

## Computer Implementations

The *k*-leaky descendants in general and some of the recursive formulas from the paper in particular have been implemented in admcycles [9]. To use the code, it’s necessary to install the latest version from

https://gitlab.com/modulispaces/admcycles

directly. The following functions are available:

- monodromy_graphs – enumerates *k*-leaky tropical covers together with their associated multiplicities
- k_leaky_descendant – computes *k*-leaky double Hurwitz descendants using tropical covers
- J_formula – provides the symbolic formulas for special cases in Theorem 1
- H_formula – calculates one-part *k*-leaky Hurwitz numbers in arbitrary genus using the recursion formula from Lemma 17

A basic example calculation is shown below:

**Figure:**
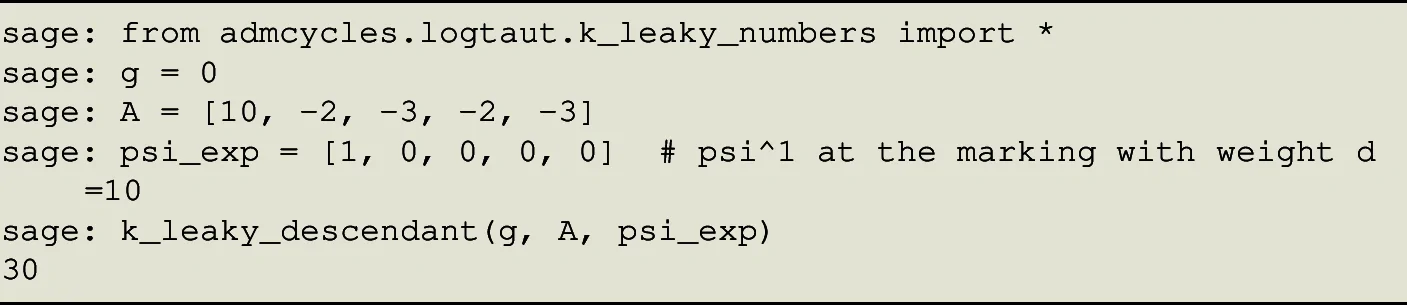

Further example calculations can be found in this notebook.

## Footnotes

1 Observe that |E(π)|=1,2,3 depending on the number of left and right edges incident to $\textrm{v}_1$.
